# Some New or Imperfectly Understood Points in the Anatomy of the Horse’s Hoof

**Published:** 1880-04

**Authors:** John A. McLaughlin

**Affiliations:** 503 Jersey Avenue, Jersey City, N. J.


### Art. VII.—SOME NEW OR IMPERFECTLY UNDERSTOOD POINTS IN THE ANATOMY OF THE HORSE'S HOOF.

By JOHN A. McLAUGHLIN, Jr., D. V. S.

(*Continued.*)

(*From the Laboratory of Comparative Anatomy and Pathology, Columbia Veterinary College, No. II.**)

### PART I.

IN its narrower sense, the term "foot," as used among veterinarians, refers to the hoof and the structures contained within it. It should be recollected however, that, in an anatomical sense, this limitation of the term is inaccurate. From the stand-point of comparative anatomy indeed, the *true foot* of the horse includes the greater length of the free portions of both the anterior and posterior extremity, and extends from the so-called knee in the former, and the hock in the latter, all the way to the ground.

The term "foot," as employed by veterinarians, is therefore as inappropriate, scientifically, as the designation "knee" instead of the carpus, and the day may not be remote when "foot" and "knee," used in this sense, will, with those evolutions of stable literature, "stifle" and "snaffle," become deservedly obsolete.

The hoof consists first, as its bony foundation of the third digit, or *Os pedis*, and of the sesamoid bone that increases the articular surface continuity of the former for the reception of the second digit; this sesamoid is commonly known as the *Os naviculare*. Both these bones are completely enclosed within the

* No. I of the monographs emanating from this laboratory, and entitled "The Peduncular Tracts of the Anthropoid Apes," was published in the *Journal of Mental and Nervous Diseases*, July, 1879.

horny case; that is, if I were to saw through the foot, just at the upper contour of the horny case, both these bones would lie entirely beneath the surface of the section, and not be struck by the saw.

In such a section, however, I perceive that I have struck another bone with my saw; on examining it more closely, I find that the instrument has divided the second digit, a portion of which, therefore, falls within the horny case, another portion above it.

It is ordinarily stated in the text-books that the lower half of the second digit falls within the horny case. This is an inexact statement. As is well known, the axis of the digital bones is far from being a vertical one; when the horse stands at rest it inclines backward so as to form an angle of about forty-two degrees with the ground. Now, the upper contour of the hoof horn, although not strictly horizontal, and sinking somewhat as we pass backwards, yet deviates comparatively little for its greater extent from the horizontal level.* It therefore follows that the axis of the second digit is oblique to the ideal plane of the upper hoof contour. Consequently *more* of its posterior surface, and *less* of its anterior surface is included in the hoof. On comparing a large number of feet, I find that about two-fifths of the anterior face and fully two-thirds of the posterior face fall below what I shall designate, for the sake of brevity, as the perioplic plane (because corresponding to the ring of the periople).

We have therefore to start with, as the bony core of the hoof structures,

1. The entire *Os Pedis.*
2. " *Os Naviculare.*
3. Part of the *Os Coronae.*

Now these bones are united by ligaments in such a way as to constitute an anatomical continuity, and as if to increase the extent of the hard parts still further, we have added the so-called complimentary fibro-cartilages.

---

* With a level ground surface it forms an angle open forwards of about twenty or twenty-five degrees.

So many erroneous notions prevail as to the relations of these structures that it may be well to describe them in detail.

The simplest conception of the lateral cartilages is to consider them as the unossified extension of the lateral parts (basilar and retrossal processes), of the Os pedis, a view that is borne out by the gradual extension of ossification, and the encroachment of the Os pedis through this ossification on the cartilaginous field. Now these lateral cartilages are not only connected with the Os pedis, of which they represent, as stated, the lateral prolongations, but also with the Os coronae. I have satisfied myself that arching over the anterior lateral, or corono-pedal ligament there is a demonstrable tissue connection or transition from the upper border of the cartilage to the periosteum of that part of the Os coronae which lies by the side of the extensor pedis tendon, and near the corona pedal articular surface.

Taking one of the laterial cartilages as a whole, it may be compared to an oblique angle parallelopipedon. Its upper and lower sides are horizontal; its anterior and posterior sides are inclined forwards; the anterior side is the one which is fused in with the postero lateral part of the *ospedis.*

Although so different in histological structure from the *ospedis*, yet morphologically it is in striking similarity to it, presenting the same peculiar vascular channels and notches characteristic of the *ospedis*, so that when ossification gradually invades it, the previously *cartilaginous* channels become *bony* channels, anastomosing with those in the body of the bone.

The lateral cartilages are of great importance (although passively so) in admitting of lateral expansion in the hoof, and one of the reasons for the diminishing expansibility of the hoof in old horses is to be sought for in the concomitant ossification of old age.

On macerating the foot, and separating the anterior superior angle of the cartilage from its attachments to the *os coronæ* which I referred to above, we notice some ligamentous fibres; these will be designated as the *corono-chrondral ligament.* It plays a role in some of those pathological processes which lead to osseous hyperplasia.

If we now imagine the three bones and the two lateral cartilages *in situ* by themselves, and examine them in this relation, as the plantar surface rests on the floor, we notice that behind, there is an immense cavity, as it were, topped by the plantar surface of the os pedis and os naviculare, and flanked by the pedal wings and lateral fibro cartilages. From this relation it follows that the cavity must be narrower and lower in front, wider and higher behind, where it is also open to the observer.

Now, imagine a wax cast made of this cavity, and this cast will require very few modifications to make it resemble the peculiar tissue which fills the gap in the living horse, and is known as the *plantar cushion.*

From the statement just made, it must be evident that the plantar cushion is massive behind, and gradually attenuated forwards, becoming ultimately reduced to a thin edge, which loses itself on the plantar surface of the ospedis immediately in front of the semilunar crest.

It has an upper surface, looking towards the postero inferior faces of the ospedis and os coronæ; a posterior surface which, reckoning only the tissues and elements so far described (for I am endeavoring to build up the foot, as it were, adding one element after another), is free, and an inferior surface, which, similarly, as far as we have gone, is also free.

One of the best text-books on equine anatomy states that the plantar cushion is that part of the "complementary apparatus" of the hoof which serves to hold the two lateral cartilages together. Now, a very brief reflection will show that the function of the plantar cushion must be anything else than that of a uniting band. From its very position and shape, a wedge, pressure on whose lower part will serve to drive the lateral cartilages apart, it must be considered as having for one of its functions the prevention of a *too close approximation* of the lateral cartilages. These, from their intimate relations with the horny heels, are subject to a continual strain inwards, which, when the beneficial counteracting influence of the plantar cushion is lost by atrophy, leads to contraction of the heels.

The plantar cushion is therefore, besides being a filling out tissue for the great gap in the bony and cartilaginous structures, and besides preventing the undue transmission of strain on the great flexor tendon and the corono-pedo-navicular articulation (in impingement of the frog), one of the most important structures in maintaining the proper proportions of the foot.

The manner in which the plantar cushion and fibro cartilages enter into the mechanism of the foot will be studied when we have completed the consideration of the microscopic tissues and their developement.

The manner in which the extensor pedis and flexor perforans tendons enter the hoof, are so well known as to require no description.

As it were analogous to a film over all the structures here enumerated, in so far as they reach the surface, there is spread out that important tissue the keratogenic membrane. Considered as a whole, it covers the lower part of the anterior surface of the os coronæ, separated from the bone proper by the extensor tendon, the outer convex surface of the os pedis, the outer surface, posterior edge, and part of the inner surface of the lateral cartilages, the entire inferior, and almost the entire posterior faces of the plantar cushion, and that anterior zone of the plantar surface of the os pedis, which is not covered by the plantar cushion.

From the different names given to the different regions of this membrane, the student is often misled into believing that there exists some fundamental difference between them. But whether the term be coronary band or coronary cushion, villous tissue, perioplic band, velvety tissue or keraphyllous tissue, all these are but different local divisions of one and the same tissue, and all of them are more or less concerned, as I shall endeavor to show, in the development of the hoof-horn.

Histologically, a modified portion of the tegumentary covering corresponding to the *derma*, plus the *rete mucosum*, it exhibits two different structural forms: the *villous tissue* and the *laminal tissue.* The laminal tissue, whose characteristic resemblance to the leaves of an opened book is familiar to all who have dissected the foot, is found on the convexity of the os pedis and cartilages, and is inflected on the inner surface of the latter around their posterior border. The individual laminæ increase in depth as we follow them from above downwards; that is, the ridges are higher towards the ground. Their length from above downwards decreases as we pass sidewards and doubling the posterior cartilaginous border reach the inner border, where they disappear by gradual diminution.

The villous tissue is more evenly spread on the plantar and posterior surface, and skirting the posterior border of the cartilages it passes over along the upper confines of the laminal tissue on the front of the foot, to constitute a thick convex band, encircling the region of the lower third of the os coronæ. It constitutes the coronary cushion or band. Everywhere the villous tissue is a powerful *primary** horn producer. The *wall* of the hoof is rooted, as it were, in the coronary band, and the frog and sole are correspondingly molded on the villous tissue covering the plantar cushion and plantar surface of the os pedis.

The wall, even in its inflections, enters into a secondary union with the laminal or the keraphyllous tissue; that is, wherever you have wall, whether it be any of the segments known as toe, outside or inside toe, outside or inside quarter, heels or bars, there you will find underneath laminal tissue.

I had hinted previously that while the plantar cushion might in a crude way be considered as a cast into the gap between the cartilages and bones, that there were some minor modifications. These minor modifications are of importance in their relation to the architecture of the hoof horn.

Thus a median eminence on the anterior part of the ground surface, known as the pyramidal eminence, corresponds to that eminence of the horn which is known as the "frog point." A depression immediately behind it receives the frog ridge, and corresponds to the median lacuna of the frog externally. Two bulbous enlargements, directed backwards and overlying the posterior border of the cartilages, and known as the bulbs of the cushion, equally correspond to the glomes or heels of the horny frog.

* To be distinguished from *secondary* horn production, which we will explain in the sequel.

A very expressive parallel for the inferior aspect of the plantar cushion would be a fore-shortened pair of scissors, whose handles were imperforate. The handles would correspond to the bulbs, the gap between them to the median lacuna, and the closed cutting blades to the pyramidal eminence.

Thus much, well known I have no doubt to the majority of the readers, I have gone over as a preliminary to the discussion of subjects which, as the sequel will show, are complex and controversial enough.

503 JERSEY AVENUE, Jersey City, N. J.

